# Uterine Expression of NDRG4 Is Induced by Estrogen and Up-Regulated during Embryo Implantation Process in Mice

**DOI:** 10.1371/journal.pone.0155491

**Published:** 2016-05-13

**Authors:** Qian Yang, Yan Gu, Xuan Zhang, Jian-Mei Wang, Ya-Ping He, Yan Shi, Zhao-Gui Sun, Hui-Juan Shi, Jian Wang

**Affiliations:** 1 NPFPC Key Lab of Reproduction Regulation, SIPPR, School of Pharmacy, Fudan University, Shanghai, 200032, China; 2 The Second Hospital of Tianjin Medical University, Tianjin, 300211, China; State Key Laboratory of Reproductive Biology, Institute of Zoology, Chinese Academy of Sciences, CHINA

## Abstract

Embryo implantation is an essential step for the establishment of pregnancy and dynamically regulated by estrogen and progesterone. NDRG4 (N-myc down-regulated gene 4) is a tumor suppressor that participates in cell survival, tumor invasion and angiogenesis. The objective of this study was to preliminarily explore the role of NDRG4 in embryo implantation. By immunohistochemistry (IHC) and quantitive RT-PCR (qRT-PCR), we found that uterine expression of NDRG4 was increased along with puberal development, and its expression in adult females reached the peak at the estrus stage during the estrus cycle. Furthermore, uterine NDRG4 expression was significantly induced by the treatment of estradiol (E_2_) both in pre-puberty females and ovariectomized adult females. Uterine expression pattern of NDRG4 during the peri-implantation period in mice was determined by IHC, qRT-PCR and Western blot. It was observed that NDRG4 expression was up-regulated during the implantation process, and its expression level at the implantation sites was significantly higher than that at the inter-implantation sites. Meanwhile, an increased expression in NDRG4 was associated with artificial decidualization as well as the activation of delayed implantation. By qRT-PCR and Western blot, we found that the *in vitro* decidualization of endometrial stromal cells (ESCs) was accompanied by up-regulation of NDRG4 expression, whereas knockdown of its expression in these cells by siRNA inhibited the decidualization process. In addition, Western blot analysis showed that NDRG4 protein expression was decreased in human villus tissues of recurrent miscarriage (RM) patients compared to normal pregnant women. Collectively, these data suggested that uterine NDRG4 expression could be induced by estrogen, and NDRG4 might play an important role during early pregnancy.

## Introduction

Emrbyo implantation is a precisely regulated step during establishment of pregnancy. Impaired implantation could cause placentation-related diseases, such as preeclampsia (PE) and recurrent miscarriages (RM) [1, 2]. The formation of uterine receptivity is an essential event for the scuccessful embryo implantation, and it has been well demonstrated that, ovarian hormones, progesterone and estrogen, control the development of uterine receptivity [3].

Uterine development occurs postnatally in an ovary- and steroid-dependent manner in many species, including rodent and human. Endometrial epithelial cells (EECs) and stromal cells (ESCs) in uterine tissues of sexually matured individual produce molecules that are essential for embryo survival and implantation[4]. A series of complex physiological events occur during the exquisitely regulated process of implantation, including extensive degradation and remodeling of the extracellular matrix, invasion of trophoblast cells into the maternal endometrium, and decidualization of ESCs[5]. Given many similarities exist between the embryo implantation and the tumorgenesis, such as establishment of an invasive phenotype, repression of specific cell adhesion molecules, elaboration of matrix-digesting enzymes, expression of proto-oncogene products, acquisition of a rich blood supply, and elude the immune system[6], molecules involved in tumorgenesis might also play critical role in embryo implantation.

Myc protein is a transcription factor that participates in the regulation of cell proliferation and differentiation, and exerts its biological functions through direct or indirect activation of its downstream regulated genes [7]. N-myc belongs to the Myc family and presents a dynamic expression pattern during mouse embryonic development [8]. N-Myc downstream-regulated gene 4 (NDRG4) is a member of the N-Myc downstream regulated gene family (NDRG). The NDRG family is highly conserved, and the identity between human NDRG4 and mouse NDRG4 is about 98% when running Blast in Pubmed. It has been reported that, NDRG4 was predominantly expressed in the early postnatal brain and involved in cell growth and proliferation [9, 10]. Meanwhile, accumulated evidences showed that NDRG4 possess tumor-suppressive and oncogenic functions depending on the tissue type [11], and might be a potential biomarker for predicting aggressive forms of cancer [12–14].

Recently, a role of NDRG2 in mouse embryo implantation was reported [15], and one of the predicted upstream-regulated microRNAs of NDRG4, miR-3074-5p, was found to be involved in embryo implantation in mice [16], thus, we hypothesized that NDRG4 might also participate in the embryo implantation, and the present study was undertaken to examine the uterine expression pattern of NDRG4 during the peri-implantation period in mice and its role in decidualization of ESCs.

## Results

### Uterine NDRG4 expression during the different postnatal development stages

**Fig 1:**
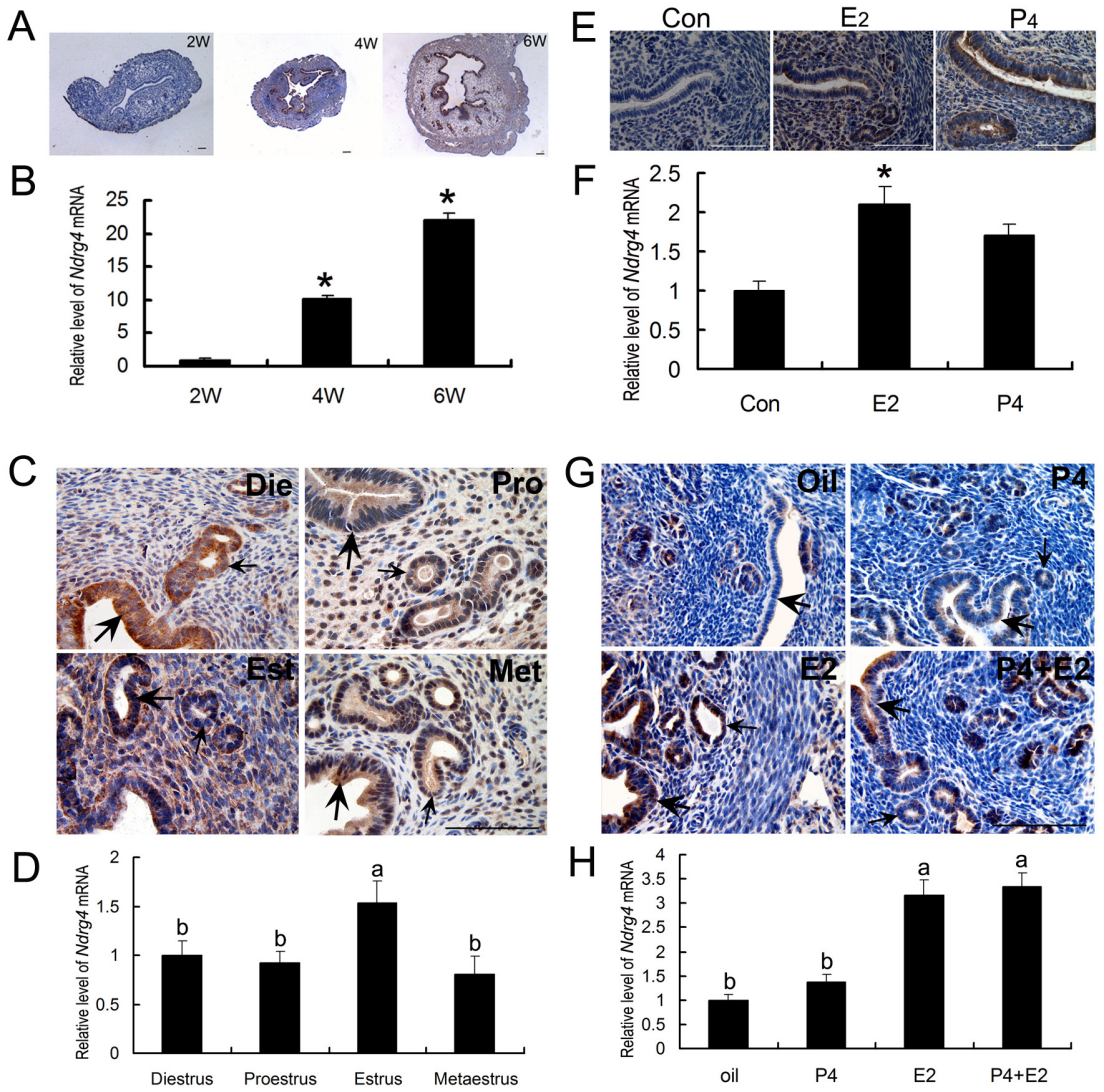
Effects of estrogen on uterine NDRG4 expression. (A) Immunohistochemical analysis of uterine NDRG4 expression during the different postnatal development stages, 2W, 2-weeks old; 4W, 4-weeks old; 6W, 6-weeks old. (B) Quantitative PCR analysis of uterine *NDRG4* mRNA expression during the different postnatal development stages (n = 3), *, significantly different (*P* < 0.05). (C) Immunohistochemical analysis of NDRG4 expression at the diestrus (Die), proestrus (Pro), estrous (Est) and metestrus (Met) phases during the estrous cycle. (D) Quantitative PCR analysis of uterine *NDRG4* mRNA expression during the estrous cycle (n = 3), the thick arrow shows the luminal epithelium, the small arrow indicates the glandular epithelium, the columns with different superscripts are significantly different (*P* < 0.05). (E) Immunohistochemical analysis of uterine NDRG4 expression in prepuberty mice respectively treated by oil (Con), estrogen (E_2_) and progesterone (P_4_). One-week-old female mice (pre-pubertal stage) were divided into 3 groups (n = 3, per group) at random, and administered daily subcutaneous injections of (1) E2, (2) P4, or (3) sesame oil, respectively, for 7 days. (F) Quantitative PCR analysis of uterine *NDRG4* mRNA expression in prepuberty mice following steroid hormone treatment (*n* = 3). *, significantly different (*P* < 0.05). (G) Immunohistochemical analysis of NDRG4 protein expression in ovariectomized mice respectively treated by oil (oil), progesterone (P_4_), estrogen (E_2_), and progesterone plus estrogen (P_4_+E_2_). (H) Quantitative PCR analysis of uterine *NDRG4* mRNA expression in ovariectomized mice following steroid hormone treatment (*n* = 3). The thick arrow indicates the luminal epithelium. The small arrow shows the glandular epithelium. The columns with different superscripts are significantly different (*P* < 0.05).

Uterine expression of NDRG4 during different postnatal development stages were determined by immunohistochemistry (IHC) and real-time quantitative RT-PCR (qRT-PCR) analyses. The results of IHC showed that there were no visible signals were found in 2-weeks old (2W) mouse uteri, whereas distinctive NDRG4 protein signals in luminal and glandular epithelial cells were found in 4-weeks (4W) and 6-weeks old mouse uteri (Fig 1A). Consistently, qRT-PCR analysis revealed that uterine expression level of *NDRG4* mRNA was significantly increased from prepuberty (2W) to puberty (6W) (Fig 1B and S1 File).

### Uterine NDRG4 expression in adult mice during the normal estrous cycle

Uterine expressions of NDRG4 during estrous cycle were determined by IHC and qRT-PCR. The results showed that, distinctive NDRG4 protein signals were detected in the diestrus (Die), proestrus (Pro), estrous (Est) and metestrus phases (Met), and mainly localized in luminal and glandular epithelial cells (Fig 1C). At the estrous phase (Est), weak positive signals of NDRG4 protein was observed in stromal cells (Fig 1C). Real-time qRT-PCR analysis revealed that, uterine expression level of *NDRG4* mRNA was significantly increased at the estrous phase compared to that at the diestrus, proestrus and metestrus phases (Fig 1D and S2 File).

### Effect of steroid hormones on uterine NDRG4 expression in mice

To examine the effect of exogenous steroid hormone on uterine NDRG4 expression in the pre-puberty mice, one-week-old female mice (pre-pubertal stage) were administered daily subcutaneous injections of E_2_ or P_4_ for 7 days. We found that NDRG4 protein signals were obviously enhanced by the treatment of E_2_ (E_2_) compared to control (Con), and were dominantly localized both in the luminal and glandular epithelia. The NDRG4 protein signals in P_4_ treatment group (P_4_) were stronger than control gourp (Con), but weaker than E_2_ treatment group (E_2_) (Fig 1E). A similar alteration in *NDRG4* mRNA expression was observed following treatment with E_2_ or P_4_ (Fig 1F and S3 File). In ovariectomized adult mice, it was observed that, NDRG4 protein signals in E_2_-treatment group (E_2_) and E_2_ plus P_4_-treatment group (P_4_+E_2_) were dominantly localized in the luminal and glandular epithelia, and were obviously stronger than that in control group (oil) and P_4_-treatment group (P_4_) (Fig 1G). Uterine expression levels of *NDRG4* mRNA expression in in E_2_-treatment group (E_2_) and E_2_ plus P_4_-treatment group (P_4_+E_2_) were significantly increased that that in control group (oil) and P_4_-treatment group (P_4_) (Fig 1H and S4 File).

### Uterine expression pattern of NDRG4 during the peri-implantation period in mice

**Fig 2:**
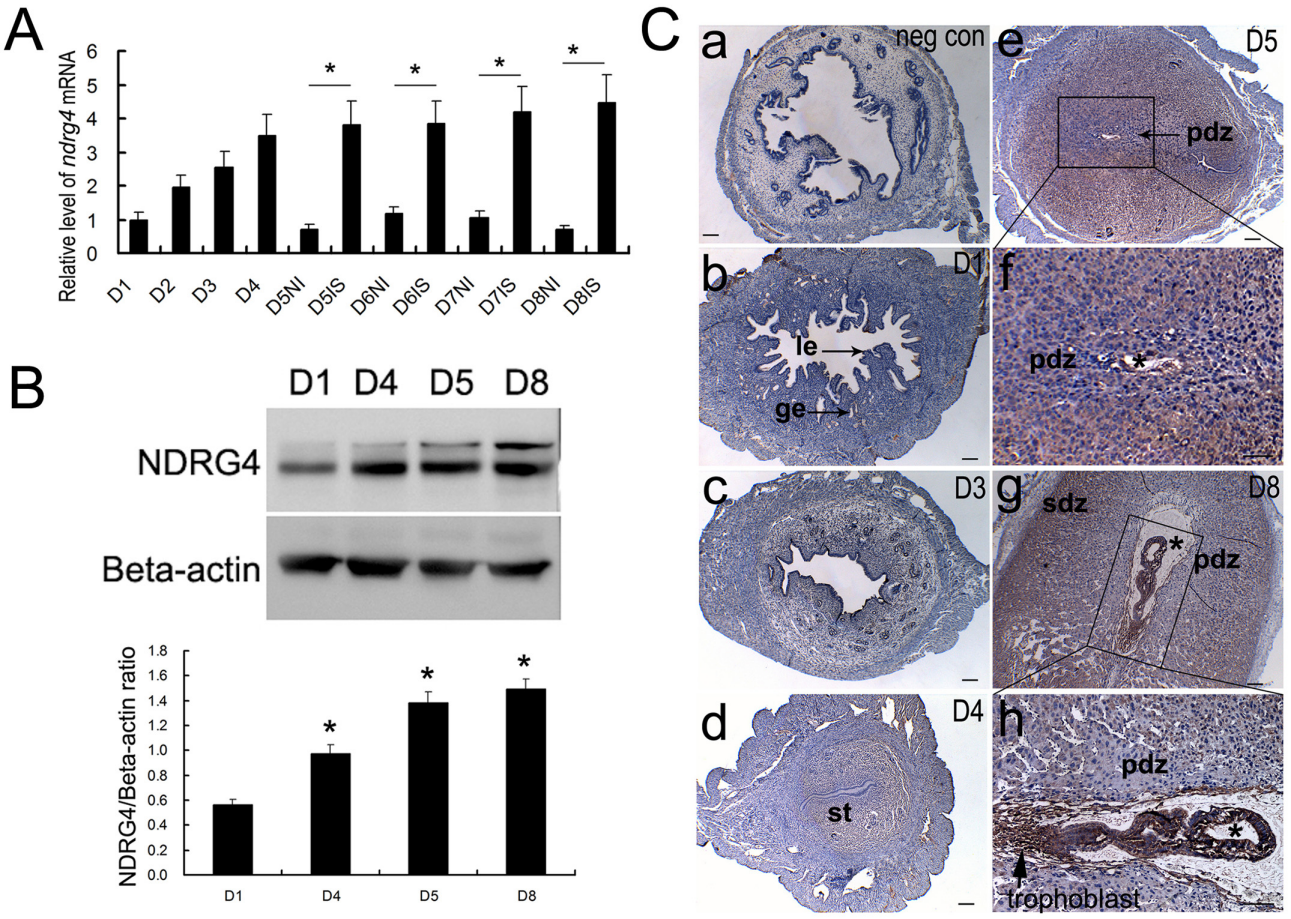
Uterine NDRG4 expression during early pregnancy in mice. (A) Quantitative PCR analysis of *NDRG4* mRNA expression in the uterus during early pregnancy (n = 3, *P < 0.05). (B) Western blot and densitometric analyses of uterine NDRG4 protein levels during early pregnancy (*n* = 3). All experiments were repeated three times. The data are shown as the mean ± SEM. *P < 0.05. (C) Immunohistochemical analysis of uterine NDRG4 protein expression on days 1, 3, 4, 5, and 8 of pregnancy. IS, implantation sites; NI, non-implantation sites. *, indicates the location of the embryo. le, luminal epithelium; ge, glandular epithelium; st, stroma; pdz, primary decidual zone; sdz, secondary decidual zone; D, day of pregnancy; gc, giant cell; Scale bar represents 100 μm.

RT-PCR results showed that, during the peri-implantation period, the uterine *NDRG4* mRNA expression level was gradually increased from day 1 (D1) to day 8 (D8) of pregnancy. Especially, during the implantation process from day 5 to day 8 of pregnancy, *NDRG4* mRNA expression level at the implantation sites (IS) was significantly up-regulated compared to that at the inter-implantation sites (Int-IS) (Fig 2A and S5 File). It was also found by Western blot analysis that, uterine NDRG4 protein expression levels on days 4, 5 and 8 of pregnancy were significantly up-regulated compared to that on days 1 of pregnancy (Fig 2B, S1 Fig and S6 File). Furthermore, the spatiotemporal expression of NDRG4 protein in the mouse uterus during early pregnancy was examined by IHC. There were weak NDRG4 protein signals in the uterine tissue obtained from pregnant mice on day 1 (Fig 2Cb) and day 3 (Fig 2Cc). On day 4 of pregnancy, faint NDRG4 protein signals were detected in the subepithelial stromal bed (Fig 2Cd). On day 5 of pregnancy, the distinct accumulation of NDRG4 protein signals was observed in the primary decidual zone (PDZ) that adjacent to the implanting embryo (Fig 2Ce and 2Cf). The distribution of NDRG4 protein signal on day 8 of pregnancy was similar to that on day 5, and the signals were observed in invaded trophoblasts at the implantation site (Fig 2Cg and 2Ch).

### Association of uterine NDRG4 expression with the artificial decidualization and activated implantation in mice

**Fig 3:**
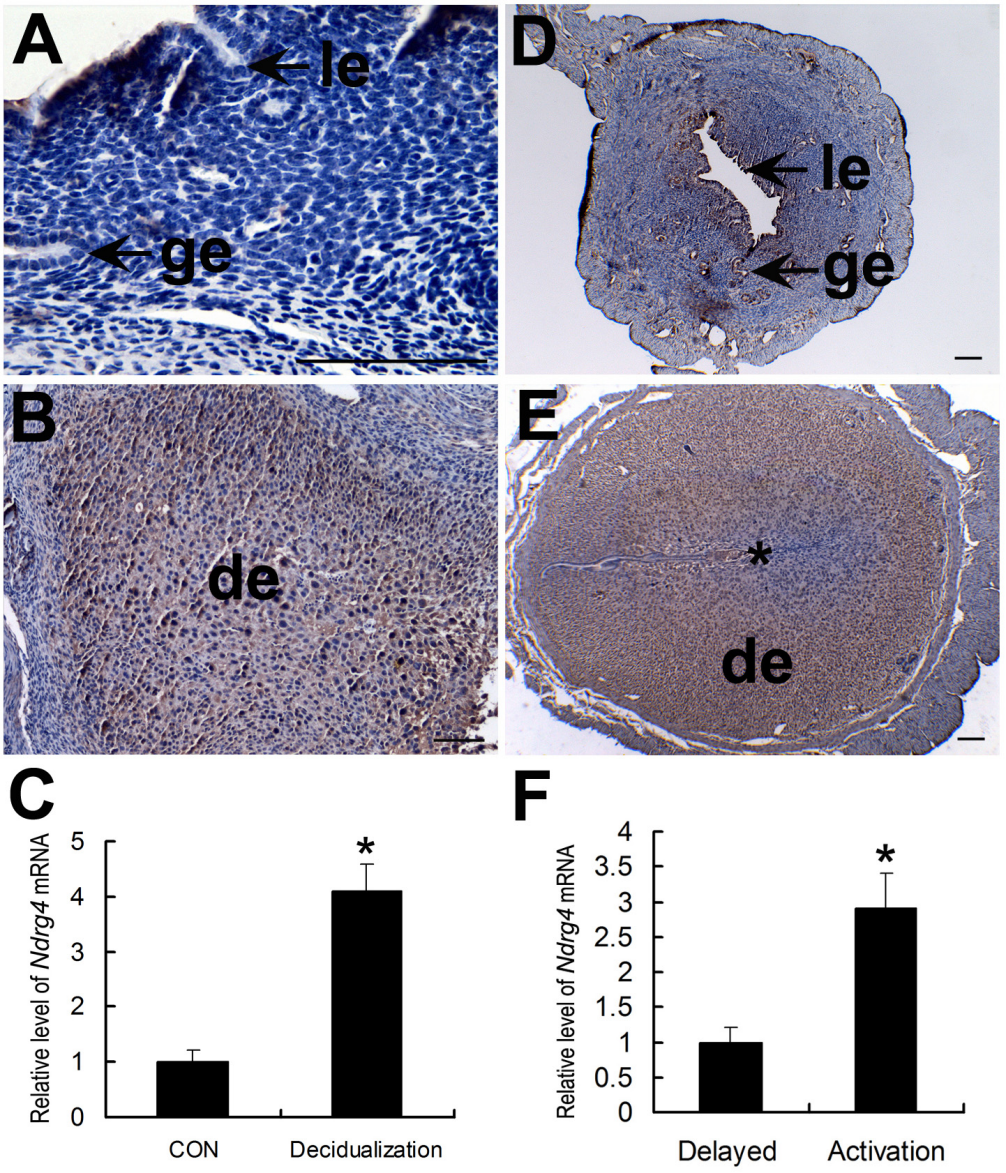
Uterine NDRG4 expression following artificial decidualization and activation of delayed implantation. Immunohistochemical analysis of uterine NDRG4 protein expression following artificial decidualization (B) and in its contralateral uninjected uterine horn (A), under delayed implantation (D) and activation (E). Quantitative PCR analysis of *NDRG4* mRNA expression in the uterus following artificial decidualization (C) and activation of delayed implantation (F) (*n* = 3). The thick arrow indicates the luminal epithelium. The small arrow shows the glandular epithelium. De, decidua; *, significantly different from control (*P* < 0.05).

The artificial decidualization model and delayed implantation model were used to examine whether uterine NDRG4 expression is induced by the decidualization reaction and/or dependent on the presence of a living embryo. The results of IHC analysis showed that, strong NDRG4 protein signals were detected in decidualized stromal cells (Fig 3B), whereas no visible signals were found in control uteri (Fig 3A). Meanwhile, uterine *NDRG4* mRNA expression level was also found to be significantly up-regulated by artificial decidualization (Fig 3C and S7 File). In the delayed implantation model, the NDRG4 protein was abundantly localized in decidual zone in the activated implantation uteri (Fig 3E), and it was weakly expressed in delayed implantation uteri (Fig 3D). Likewise, *NDRG4* mRNA expression level in the activated implantation uteri was significantly up-regulated compared to that in the delayed uteri (Fig 3F and S8 File).

### NDRG4 expression in cultured mouse ESCs during the process of *in vitro* decidualization

**Fig 4:**
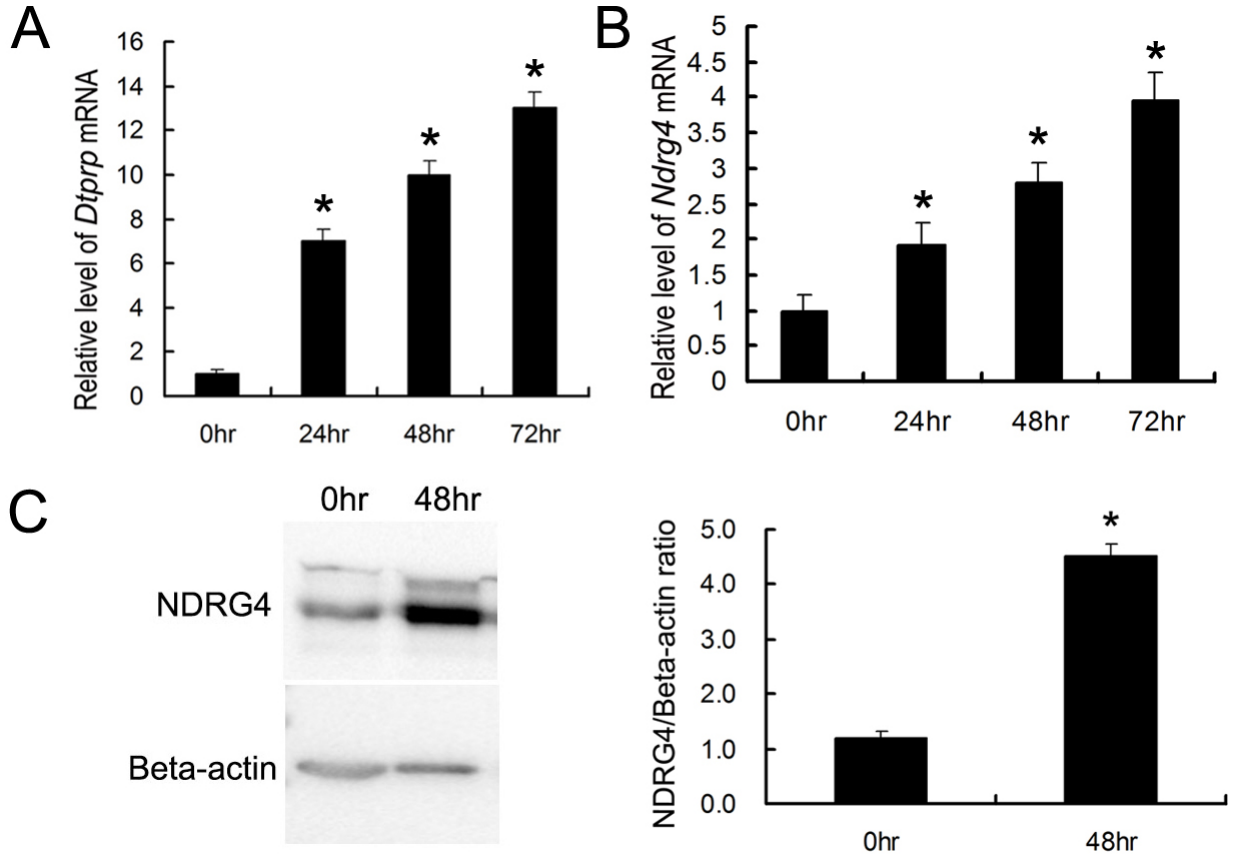
*In vitro* decidualization of mouse primary ESCs. (A) qRT-PCR analysis of *DTPRP* mRNA expression in ESCs cultured for up to 72 h. (B) qRT-PCR analysis of *NDRG4* mRNA expression in cultured ESCs. (C) Western blot analysis of NDRG4 protein expression in ESCs cultured for up to 48 h. Densitometric analyses of NDRG4 compared with 0 h is shown. The values represent the mean ± SEM, as determined from three separate experiments. *, significantly different (*P* < 0.05).

To explore the function of NDRG4 in the decidualization of mouse ESCs, a mouse primary ESCs culture system was established according to previously published method [15]. The transformation of decidual cells from ESCs induced by E_2_ and P_4_ was indicated by the expression of decidual/trophoblast PRL-related protein *(DTPRP)* mRNA[17] (Fig 4A and S9 File). Expression levels of NDRG4 protein and *NDRG4* mRNA in ESCs were found to be significantly increased during the process of *in vitro* decidualization (Fig 4B and 4C, S2 Fig, S10 and S11 Files).

### Effects of down-regulated NDRG4 expression in ESCs on *in vitro* decidualization

**Fig 5:**
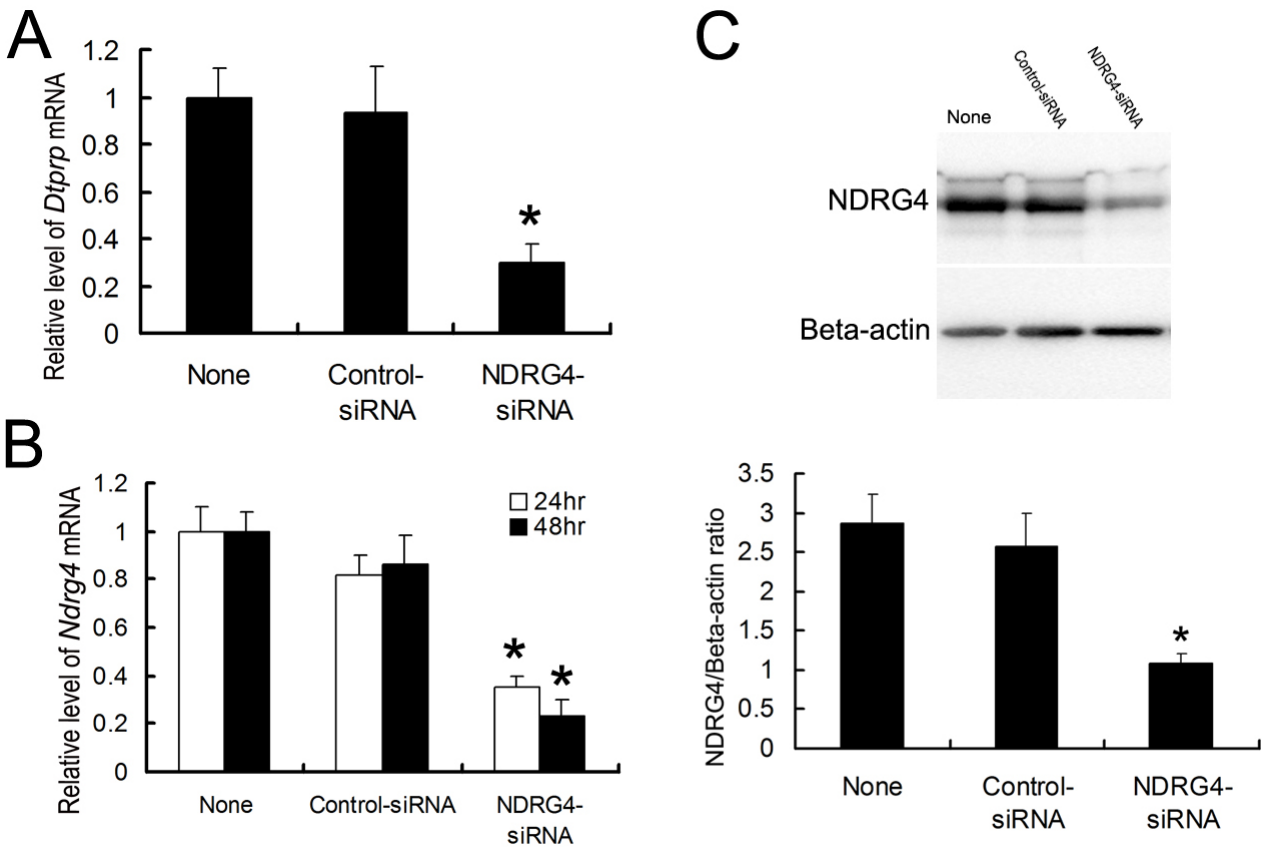
Down-regulation of NDRG4 expression in ESCs inhibits *in vitro* decidualization. Quantitative PCR analyses of *DTPRP* mRNA expression (A) and *NDRG4* mRNA expression (B) in ESCs transfected with *NDRG4*-targeting siRNAs or non-targeting siRNAs at 24 h and 48 h. Western blot and densitometric analyses of NDRG4 protein levels in ESCs at 72 h after transfection (C). The relative fold induction of NDRG4 protein expression compared with its expression in non-siRNA-treated group is shown. The values represent the mean ± SEM, as determined from three separate experiments. *, significantly different (*P* < 0.05).

To further explore the role of NDRG4 in the decidualization of ESCs, NDRG4 expression in cultured ESCs was knocked down using its targeting siRNAs. As a result, expression of NDRG4 was reduced by more than 60% in *NDRG4* siRNA-transfected ESCs (NDRG4-siRNA) compared with that in non-transfected ESCs (None) and control siRNA-transfected ESCs (Control-siRNA) (Fig 5B and 5C, S2 Fig, S13 and S14 Files), and this reduction was correlated with a significant decrease in *DTPRP* mRNA expression (Fig 5A and S12 File), indicating that down-regulation of NDRG4 expression inhibited the decidualization of mouse ESCs.

### Expression of NDRG4 in human villus tissues of RM patients and normal pregnancy women

**Fig 6:**
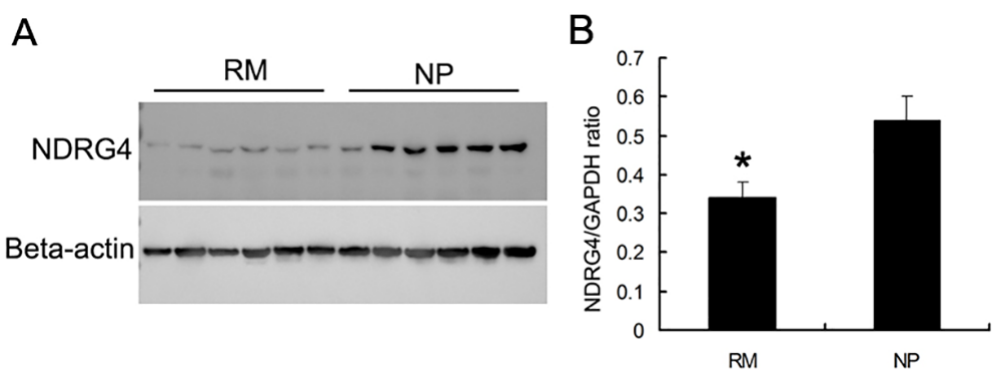
NDRG4 expression in human villus tissues of RM patients and normal pregnant (NP) women. Western blot analysis of NDRG4 protein expression (A) and densitometric analyses of NDRG4 protein in villus tissues of RM patients and normal pregnant women (B). *, significantly different (*P* < 0.05).

To preliminarily explore that whether or not the data derived from the mouse model might be to extend to human, we collected 6-pair villus tissues of RM patients and normal pregnant (NP) women. By Western Blot, we found that, the NDRG4 protein expression level in villus tissues of RM patients was significantly decreased compared to that in NP women (Fig 6, S15 File and S3 Fig).

## Discussion

In the present study, we demonstrated the uterine expression pattern of NDRG4 during different postnatal development, the estrous cycle and early pregnancy in mice. The uterine NDRG4 expression level was increased along with the mouse puberal development, and its expression in adult females peaked at the estrus stage. Exogenous estrogen could induce the uterine expression of NDRG4 both in pre-puberty females and ovariectomized adult females. During early pregnancy, uterine NDRG4 expression was significantly increased at implantation sites, with predominant localization in the decidual zone and invaded trophablasts. The increase of NDRG4 expression was accompanied by the activation of delayed implantation, as well as artificially induced decidualization both *in vivo* and *in vitro*. Furthermore, down-regulation of NDRG4 expression in mouse ESCs significantly inhibited decidualization *in vitro*. The significant decrease in expression of NDRG4 in human villus tissues of RM patients was also observed.

Uterine development occurs postnatally in an ovary- and steroid-independent manner, and the synergistic actions of E_2_ and P_4_ are critical for regulation of the estrous cycle and establishment of uterine receptivity [4, 18]. It was found in the present study that, uterine NDRG4 expression was significantly increased after E_2_ administration, and during the normal estrous cycle, uterine NDRG4 expression was significantly increased at the estrous stage of which E_2_ surge takes place [19], indicating a stimulating effect of estrogen on the uterine expression of NDRG4 in mice. Estrogen is involved in a variety of reproductive functions. Mammalian endometrium maintain regular estrous cycle and achieve an optimal uterine environment for embryo implantation through estrogen receptor (ER) pathway. In estrogen classical pathway, the activated ER binds to estrogen response elements (EREs) of the target gene to regulate its expression. However, it has been demonstrated that estrogen could regulate gene expression through the nonclassical pathway, in which the activated ER interacts with transcription factors activator protein-1 (AP-1) [20]. It has been reported that AP-1 binding site was a candidate regulatory element in NDRG4 molecule [21]. As NDRG4 protein was detected in endometrial epithelial cells and stromal cells, we speculated that it might participate in regulating cyclic proliferation and differentiation in endometrial epithelial/stromal cells to optimally prepare for embryo implantation under the control of E_2_ through nonclassical ER/AP-1 pathway during the estrous cycle.

Successful embryo implantation is integral to the establishment of pregnancy, and initiation of implantation coincides with the establishment of uterine receptivity and the subsequent decidualization of ESCs[22]. In the mouse, a pre-ovulatory E_2_ surge stimulates the proliferation of endometrial epithelial cells (EECs) on day 1 of pregnancy, followed by a rise in the P_4_ level to initiate the proliferation of ESCs on day 3. Embryos enter the uterus at midnight on day 3 or in the early hours of day 4 after the uterus is exposed to an increased concentration of P_4_ for at least 24 h on day 3 followed by exposure to estrogen, causing it to be receptive for embryo implantation [23]. Given during the pre-implantation period from day 1 to day 4 of pregnancy, uterine NDRG4 expression level sustainedly increased, suggesting a potential role of NDRG4 in establishing uterine receptivity under the regulation of estrogen.

Once mouse embryos attach to a receptive endometrium, decidualization is triggered by extensive proliferation and differentiation of ESCs into decidual stromal cells (DSCs) at implantation sites. Because the decidual response can be induced in a reproducible manner in the absence of an embryo and decidual zones are easily discernable, the mouse is a good model to investigate the mechanism of decidualization. In the mouse, at least three factors may be necessary for normal decidualization, including E_2_, P_4_ and embryonic or physical stimulation (by intra-luminal infusion of oil or scratching with a needle). Increases in E_2_ and P_4_ stimulate the proliferation and differentiation of ESCs surrounding invading trophoblast cells to support the decidualization process [23–25]. In the present study, a significant increase in NDRG4 expression was observed at implantation sites from day 5 to day 8 of pregnancy, and NDRG4 protein signals were predominantly localized to the decidual zone and invading trophoblast cells. Moreover, NDRG4 expression in uterine tissues and ESCs could be induced by artificial decidualization, and down-regulated expression of NDRG4 in ESCs by *NDRG4*-targeting siRNAs was found to remarkably inhibit decidualization *in vitro*, indicating that NDRG4 might participate in decidualization process during the early pregnancy.

Although the exact role of NDRG4 in regulating ESCs and DSCs needs to be further explored, it has been reported that NDRG4 was a novel oncogenic protein and p53 associated regulator of apoptosis in malignant meningioma cells [26]. Differentiation of ESCs into DSCs is crucial for optimal endometrial receptivity. Apoptosis in the human endometrium plays an essential role for endometrial receptivity and early implantation. It has been suggested the pro-survival signaling pathways Erk1/2 as key regulators of the sensitivity of human ESCs for death receptor-mediated apoptosis during implantation [27]. Previous results suggested that, ERK1/2 and PI3K/Akt, as p53 pathway members, played a role in the cell cycle regulation of ESCs [28], and the rapid activation of intracellular signalling cascades ERK1/2 and PI3K/Akt by growth factors and estrogens was involved in the migration of normal ESCs[29]. Meanwhile, it was reported that NDRG4 might play a role in supporting the activation of Erk [30]. Thus, it was reasonable to believe that the function of NDRG4 in ESCs and DSCs might be partially regulated by the p53 signaling pathway(s). In addition, it has been found that Erk1/2 were widely expressed throughout early-stage embryos and involved in the trophoblast development [31]. And in the present study, the NDRG4 expression was also observed in trophoblast cells at implantation sites on day 8 of pregnancy, we hypothesize that NDRG4 might also be involved in the invasion of trophoblast cells during early pregnancy.

It was recently reported that the expression level of miR-3074-5p at the implantation sites was significantly down-regulated compared to that at the inter-implantation sites, and its expression level was remarkably decreased in villus tissues of RM patients compared to that in normal pregnant women. Given NDRG4 is one of the predicted targets of miR-3074-5p, and we supposed that NDRG4 might be participate in the invasion of trophoblasts, and we therefore examined the expression of NDRG4 in human villus tissues of RM patients and normal pregnant women. It was found that the expression level of NDRG4 protein was significantly decreased in RM patients. Although this preliminary data are encouraging, the sample number was too small to guarantee the confidence. Thus, next investigation would be carried out based on a larger number of human decidual and villus specimens collected from normal pregnant women and RM patients or PE patients, with a view to futher explore the potentiality of NDRG4 to be a novel biomarker for predicting impaired implantation and placentation-related diseases.

In conclusion, the present study has demonstrated that estrogen stimulated the uterine NDRG4 expression and that NDRG4 expression level was significantly up-regulated at implantation sites during early pregnancy in mice. And an increased expression of NDRG4 was associated with the decidualization and down-regulation of NDRG4 expression in ESCs inhibited *in vitro* decidualization of ESCs. The NDRG4 expression level was significantly decreased in villus tissues of RM patients. These results suggest that NDRG4 might play critical roles in embryo implantation under the regulation of estrogen. Further investigation to study the precise molecular mechanism of NDRG4 will lead to a better understanding of embryo implantation and hopefully, the information obtained from mouse could be extrapolated to humans.

## Materials and Methods

### Animals and tissue preparation

Adult ICR mice aged 8–10 weeks were obtained from the SIPPR/BK Laboratory Animal Company (Shanghai, China) and were caged at a controlled temperature (22°C) under a 14 h light: 10 h dark photoperiod. All experiments were conducted in full compliance with standard laboratory animal care protocols that were approved by the Institutional Animal Care Committee of Shanghai Institute of Planned Parenthood Research (Approval: 2013–19). The estrous cycle was staged by examining vaginal smears as previously described[32], and subsequently the uterine horns were removed from adult females at the diestrus, proestrus, estrous, and metestrus stages (n = 3, per stage) immediately after they were sacrificed by cervical dislocation.

To observe the effects of ovarian steroid hormones on uterine NDRG4 expression in mice, uterine tissues were collected from female mice at ages of 2-weeks, 4-weeks and 6-weeks, respectively (n = 3, per stage). One-week-old female mice (pre-pubertal stage) were divided into 3 groups (n = 3, per group) at random, and administered daily subcutaneous injections of (1) 17β-estradiol (E_2_, 8 ng/g body weight; Sigma, St. Louis, MO), (2) progesterone (P_4_, 50 μg/g body weight; Sigma), or (3) sesame oil (0.1 ml/mouse), respectively, for 7 days as previously reported [33, 34]. Adult female mice were anaesthetized with pentobarbital (40mg/kg) and laparotomies were performed to remove bilateral ovary. Then the ovariectomized female mice were moved to a warm stage until they recovered from anesthesis. Then ovariectomized female mice were allowed to rest for 2 weeks. Then, the ovariectomized mice were randomly divided into four groups (n = 3, per group) and injected with (1) sesame oil (0.1 ml/mouse), (2) E_2_ (4 ng/g body weight), (3) P_4_ (40 μg/g body weight), or (4) E_2_ (4 ng/g body weight) plus P_4_ (40 μg/g body weight) according to previously described methods [35, 36]. Steroids were dissolved in sesame oil and injected subcutaneously at the same volume (0.1 ml/mouse). The mice were sacrificed by cervical dislocation at 24 h after treatment, and the uterine horns were collected.

To determine the uterine expression pattern of NDRG4 during early pregnancy, adult female mice were mated with fertile males of the same strain to achieve pregnancy (day 1 = day of vaginal plug). Pregnancy was confirmed on days 1 and 4 by recovering embryos from the reproductive tracts. Trypan blue dye solution (0.1% in saline (w/v), 0.1 ml per mouse, Sigma) was injected via the tail vein on day 5 to visualize the implantation sites. The mice were sacrificed by cervical dislocation and the entire uterine horn was collected from the pregnant mice on days 1 and 4 of pregnancy (n = 3, per day). Uterine tissues at the implantation sites (IS) and inter-implantation sites (int-IS) were separately collected from the pregnant mice on days 5 to 8 of pregnancy (n = 3, per day).

Pseudopregnant mice were obtained by mating adult females with vasectomized adult males. Vasectomy was performed as previously described [37]. Briefly, adult male mice were anaesthetized with pentobarbital (40mg/kg). The bilateral vas deferens were cut in two points at once with a micro dissecting serrated forceps, and the muscle and skin were sutured. Then the vasectomized male mice were moved to a warm place until they recovered from anesthesis. The vasectomized male would be ready to mate 2 weeks after surgery. To examine implantation, female mice were mated with vasectomized males to induce pseudopregnancy. The day of observation of a vaginal plug was considered to be day 1 of pseudopregnancy. Artificial decidualization was induced by intraluminally infusing 25 μl of sesame oil into one uterine horn on day 4 of pseudopregnancy mice (n = 3) after anaesthetized with pentobarbital (40mg/kg), and the contralateral un-injected horn served as a control (n = 3). Mice were sacrificed by cervical dislocation at 72 h after decidualization was artificially induced [38]. Decidualization was confirmed by both weighing the uterine horns and histological examination of the uterine sections.

In the delayed embryo implantation model, pregnant female mice were bilaterally ovariectomized under ether anesthesia at 08:30–09:00 h on day 4 of pregnancy. The animals in the delayed embryo implantation group and activation group were subcutaneously injected with P_4_ (1 mg/25 g body weight) dissolved in corn oil at 10:00 h from days 4 through 7 of pregnancy to maintain delayed implantation. Then, the animals in the activated implantation group (n = 3) received E_2_ (25 ng/25 g body weight) along with P_4_ to activate embryo implantation [38, 39]. The female mice were euthanized at 10:00 h on day 8 of pregnancy, and the embryos were verified as previously described [40]. Delayed or activated implantation was confirmed by microscopic observation of the metabolically dormant or activated blastocyst in the uterine flush, as previously described [38].

### Patients and tissues

All participants in this study were recruited from June 2013 to August 2013 at the outpatient department of Gynecology and Obstetrics, The Second Hospital of Tianjin Medical University, China. Trying to avoid the disturbance of confounding factors on subsequent analyses, all participants were recruited according to the same inclusion and exclusion criteria. Six RM patients (age: 29.67 ± 2 years and gestational age at sampling 7.5 ± 0.67 weeks (mean ± S.D.)), who had experienced at least two consecutive embryonic losses before the 12th gestational week and whose current pregnancy loss was objectively confirmed by transvaginal ultrasound exam, were recruited in the RM group. All clinical summaries about their personal history for thromboembolic disease and successful pregnancy or previous pregnancy losses were obtained. Classical risk factors such as abnormal parental karyotypes, uterine anatomical abnormalities, infectious diseases, luteal phase defects, diabetes mellitus, thyroid dysfunction and hyperprolactinemia were excluded by medical examinations. Meanwhile, six clinically normal pregnant (NP) women (age: 28.17 ± 4.16 years and gestational age at sampling 6.67 ± 0.44 weeks (mean ± S.D.)) (Table 1), which were terminated for non-medical reasons and undergoing legal abortions around the 6th-12th gestational week were recruited in the normal pregnancy group as the control. They were also checked for classical risk factor for pregnancy loss. After informed consent was obtained, villus tissues were collected by curettage from these 12 participants respectively. This study was approved by the Medical Ethics Committees of Shanghai Institute of Planned Parenthood Research (Ref # 2013–7, 2013–12). Written informed consents were obtained from all patients who provided tissue samples, and we have also obtained consents to publish research data derived from these collected samples.

**Table 1:** Information about recruited 6 RM patients and 6 normal pregnant women.

Group	Sample No	Age	Gestational weeks	Childbearing history	Spontaneous abortion history
**RM**	1	28	8	0	4
	2	27	6	1	3
	3	30	8	0	2
	4	28	7	0	3
	5	32	8	0	2
	6	33	8	1	3
			**Mean ± S.D.**		P *
	**Age**		29.67 ± 2		> 0.05
	**gestational weeks**		7.5 ± 0.67		> 0.05
**Normal Pregnancy**	C1	31	6	1	2
	C2	29	7	0	1
	C3	27	7	1	2
	C4	26	6	1	2
	C5	19	7	0	2
	C6	37	7	1	3
			**Mean ± S.D.**		
	**Age**		28.17 ± 4.16		
	**gestational weeks**		6.67 ± 0.44		

(*, compared to that of normal pregnancy)

### Primary culture of endometrial stromal cells and induction of decidualization *in vitro*

The isolation and culture of mouse ESCs was performed following previously described methods with minor modifications [41]. In summary, uterine horns were collected from pregnant mice on day 4 and cleaned to remove fat tissues. They were then slit longitudinally and washed thoroughly in Hanks’ balanced salt solution (HBSS, Invitrogen, Carlsbad, CA) containing 100 U/ml penicillin (Invitrogen) and 100 μg/ml streptomycin (Invitrogen). Next, the tissues were placed in HBSS containing 10 mg/ml trypsin (Sigma), 6 mg/ml dispase (Invitrogen), 100 U/ml penicillin and 100 μg/ml streptomycin for 1 h on ice, followed by incubation for 1 h at room temperature and 10 min at 37°C. Following the digestion steps, the tissues were gently mixed, and the supernatant was discarded to remove the endometrial epithelial clumps. The partially digested tissues were then washed twice in HBSS and placed into HBSS containing 0.15 mg/ml collagenase (Invitrogen) at 37°C for 30 min. Following digestion and shaking, the contents of the tube were passed through a 70 μm gauze filter (Millipore, Darmstadt, Germany) to eliminate epithelial sheets. The cell pellets were washed twice and added to Dulbecco’s modified Eagle’s Medium-F12 medium (DMEM/F12) containing 10% charcoal-stripped fetal calf serum (FBS, Invitrogen) and antibiotics at 2×10^5^ cells per well in a 6-well cell culture plate (Invitrogen). After incubation for 1 h, unattached cells were removed by several washes with HBSS, and cell culturing was continued after the addition of fresh DMEM/F12 containing 1% charcoal-stripped FBS, 100 U/ml penicillin, 100 μg/ml streptomycin, 10 nM E_2_, and 1 μM P_4_ to induce decidualization of the ESCs.

### siRNA transfection

*NDRG4*-targeting siRNAs were purchased from Santa Cruz Biotechnology, Inc. (*NDRG4* siRNA (m) sc-149865, and sc-37007 was used as the irrelevant control siRNA, Santa Cruz, Santa Cruz, CA). Prior to the *in vitro* decidualization of ESCs, *NDRG4* siRNAs and control siRNAs were transfected into cultured ESCs according to the siPORT^™^ NeoFX^™^ protocol (Ambion/Life Technologies, Grand Island, NY). Briefly, 4 μl of siPORT NeoFX transfection reagent was mixed with 100 nM of siRNA duplexes to form complexes, and this mixture was then dispersed into each well of a 6-well cell culture plate.

### Immunohistochemistry analysis

Uterine tissues were fixed in freshly prepared 4% buffered paraformaldehyde in phosphate-buffered saline (PBS) at 4°C for over 40 h. Then, the tissues were dehydrated in graded alcohol and embedded in paraffin (Leica, Wetzlar, Hessen, Germany). Sections of uteri were processed for immunohistochemical detection. Briefly, the sections (5 m) were deparaffinized and rehydrated in xylene and a graded series of ethyl alcohol, respectively, and then rinsed in PBS. Antigen retrieval was performed by placing the slides in boiling citric acid buffer (10 mmol/l of citrate sodium and 10 mmol/l of citric acid) for 15 min. The sections were cooled to room temperature and sequentially incubated at room temperature with 3% hydrogen peroxide (H_2_O_2_) in methanol for 15 min to quench endogenous peroxidases. The sections were then incubated with a rabbit anti-NDRG4 primary antibody (H00065009-M01, Abnova, Taiwan) overnight at 4°C. After being washed with PBS, the sections were incubated with a biotin-conjugated donkey anti-rabbit secondary antibody (1:200 in blocking solution, Proteintech Company, Wuhan, China). After another wash in PBS, they were incubated with peroxidase-conjugated streptavidin (1:200 in blocking solution, Proteintech Company) for 2 h. Then, they were stained with DAB (Zhongshan Corp., Beijing, China) according to the manufacturer’s protocol and counterstained with hematoxylin (Sigma). For the negative controls, 10% donkey serum was used instead of primary antibody. All the sections were examined and photographed under a microscope (DFC420C, Leica).

### Real-time quantitative RT-PCR analysis

Total RNA was extracted from uterine tissues or ESCs using TRIzol reagent (Invitrogen) according to the manufacturer’s instructions. Extracted RNA was dissolved in diethylpyrocarbonate (DEPC, Sigma)-treated water, and the RNA concentration and purity were estimated by measuring absorbance at 260 and 280 nm with a NanoDrop 2000 (Thermo Scientific, Waltham, MA). cDNAs was synthesized using M-MLV reverse transcriptase (Promega, Madison, WI), according to the manufacturer’s instructions. Real-time PCR was performed in a 20 μl reaction volume using an ABI 7500 thermal cycler (Applied Biosystems, Foster City, CA). The thermal cycling conditions were 95°C for 30 sec, followed by 40 cycles at 94°C for 5 sec and 60°C for 30 sec. Melt curve analysis and agarose gel electrophoresis were then conducted to monitor the purity of the PCR products. Beta-actin was used as an endogenous control. The following primers were used: *NDRG4*, sense, 5′-CGTGATTGGCATTGGAGTGG-3′, antisense, 5′-AGCTCCGTGTTGTTCACCAG-3′; decidual/trophoblast PRL-related protein *(DTPRP)*, sense, 5′-AAGAATGCCCTTCAGCGAGC-3′, antisense, 5′-AGCTGGTGGGTTTGTGACAT-3′; and beta-actin, sense, 5′-GGCTGTATTCCCCTCCATCG-3′, antisense, 5′-CCAGTTGGTAACAATGCCATGT-3′.

### Western blotting

The collected mouse uterine tissues and human villus tissues were quickly frozen in liquid nitrogen and granulated into a fine powder. The tissue powder was homogenized in lysis buffer (Beyotime, China). Then, the tissue lysate was centrifuged, and the supernatant was transferred into a new tube. Cultured ESCs were collected in lysis buffer, and the lysate was centrifuged to collect the supernatant. Protein concentrations were measured by Bradford assay (Bio-Rad, Hercules, CA), and 50 μg of total protein was separated on a 12% acrylamide gel and then transferred electrophoretically onto nitrocellulose membranes (Millipore). The membranes were incubated overnight at 4°C with specific primary antibodies against NDRG4 and beta-actin (Santa Cruz) or GAPDH (Santa Cruz), followed by incubation with the appropriate secondary antibodies. The blot was developed using a PhosphaGLO AP Substrate Kit (KPL, Gaithersburg, MD) according to the manufacturer’s protocol. All samples were analyzed by Western blot in triplicate. Band intensities were quantified by densitometry using ImageJ software (U.S. National Institutes of Health, MD).

### Statistical analysis

All values were presented as the mean ± SEM, as determined from at least three independent experiments. Statistical significance was assessed by one-way ANOVA. A *P* < 0.05 was considered statistically significant. Statistical analysis was conducted using SPSS 19.0 software (SPSS Software, Chicago, IL).

## Supporting Information

S1 Fig Western blot analyses of uterine NDRG4 protein levels during early pregnancy. (TIF)

S2 Fig Western blot analysis of NDRG4 protein expression in ESCs cultured for up to 48 h and NDRG4 protein levels in ESCs at 72 h after siRNA transfection. (TIF)

S3 Fig Western blot analysis of NDRG4 protein expression in villus tissues of RM patients and normal pregnant women. (TIF)

S1 File Quantitative PCR analysis of uterine *NDRG4* mRNA expression during the different postnatal development stages. (XLS)

S2 File Quantitative PCR analysis of uterine *NDRG4* mRNA expression during the estrous cycle. (XLS)

S3 File Quantitative PCR analysis of uterine *NDRG4* mRNA expression in prepuberty mice following steroid hormone treatment. (XLS)

S4 File Quantitative PCR analysis of uterine *NDRG4* mRNA expression in ovariectomized mice following steroid hormone treatment. (XLS)

S5 File Quantitative PCR analysis of *NDRG4* mRNA expression in the uterus during early pregnancy. (XLS)

S6 File Densitometric analyses of uterine NDRG4 protein levels during early pregnancy. (XLS)

S7 File Quantitative PCR analysis of *NDRG4* mRNA expression in the uterus following artificial decidualization. (XLS)

S8 File Quantitative PCR analysis of *NDRG4* mRNA expression in the uterus of delayed implantation and activation of delayed implantation. (XLS)

S9 File Quantitative PCR analysis of *DTPRP* mRNA expression in ESCs cultured for up to 72 h. (XLS)

S10 File Quantitative PCR analysis of *NDRG4* mRNA expression in cultured ESCs. (XLS)

S11 File Densitometric analyses of NDRG4 protein expression in ESCs cultured for up to 48 h. (XLS)

S12 File Quantitative PCR analyses of *DTPRP* mRNA expression in ESCs transfected with *NDRG4*-targeting siRNAs or non-targeting siRNAs. (XLS)

S13 File Quantitative PCR analyses of *NDRG4* mRNA expression in ESCs transfected with *NDRG4*-targeting siRNAs or non-targeting siRNAs at 24 h and 48 h. (XLS)

S14 File Densitometric analyses of NDRG4 protein levels in ESCs at 72 h after transfection. (XLS)

S15 File Densitometric analyses of NDRG4 protein in villus tissues of RM patients and normal pregnant women. (XLS)
