# Supplementary figures and images for: Uterine Expression of NDRG4 Is Induced by Estrogen and Up-Regulated during Embryo Implantation Process in Mice
Source: PLoS One. 2016 May 13;11(5):e0155491. doi: 10.1371/journal.pone.0155491 (PMC4866685; doi:10.1371/journal.pone.0155491)

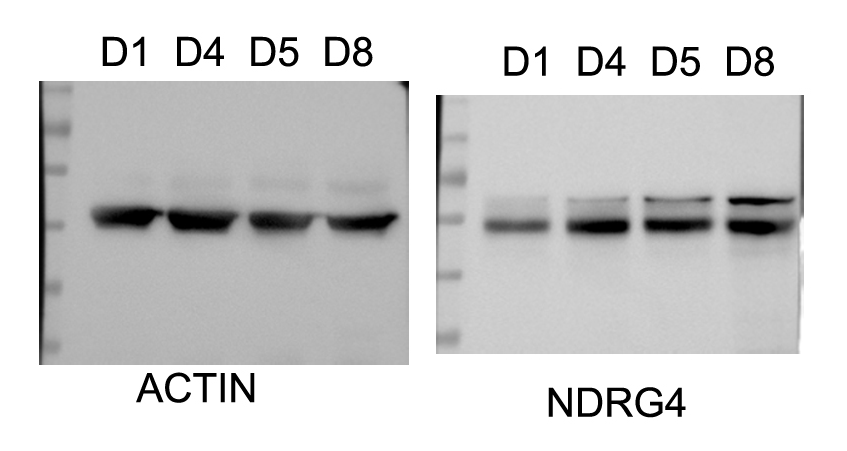

Supplement: S1 Fig — (TIF) [file pone.0155491.s001.tif]

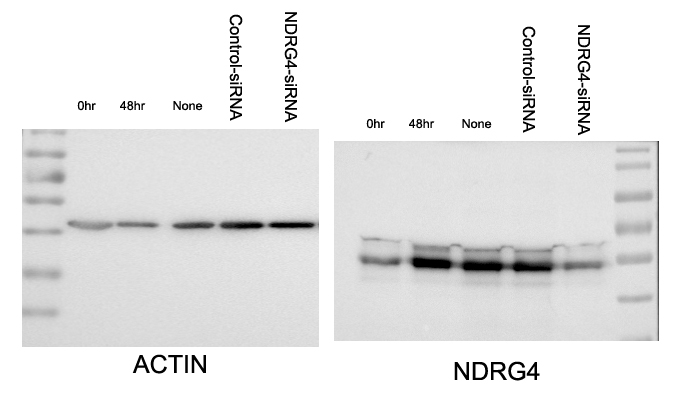

Supplement: S2 Fig — (TIF) [file pone.0155491.s002.tif]

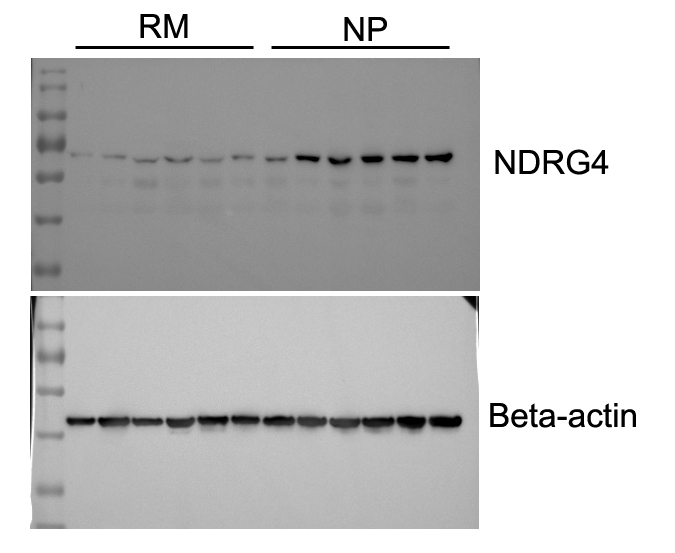

Supplement: S3 Fig — (TIF) [file pone.0155491.s003.tif]
